# Relapse patterns after radiochemotherapy of glioblastoma with FET PET-guided boost irradiation and simulation to optimize radiation target volume

**DOI:** 10.1186/s13014-016-0665-z

**Published:** 2016-06-24

**Authors:** Marc D. Piroth, Norbert Galldiks, Michael Pinkawa, Richard Holy, Gabriele Stoffels, Johannes Ermert, Felix M. Mottaghy, N. Jon Shah, Karl-Josef Langen, Michael J. Eble

**Affiliations:** Department of Radiation Oncology, University Hospital RWTH Aachen, Aachen, Germany; Department of Nuclear Medicine, University Hospital RWTH Aachen, Aachen, Germany; Department of Neurology, University Hospital RWTH Aachen, Aachen, Germany; Institute of Neuroscience and Medicine, Research Center Jülich, Jülich, Germany; Jülich-Aachen Research Alliance (JARA) – Section JARA-Brain, Research Center Jülich, Jülich, Germany; Department of Neurology, University of Cologne, Cologne, Germany; Department of Radiation Oncology, HELIOS University Hospital Wuppertal, Witten/Herdecke University, Wuppertal, Germany

**Keywords:** Glioblastoma, Radiochemotherapy, FET-PET, Relapse patterns, Target volume definition

## Abstract

**Background:**

O-(2-18 F-fluoroethyl)-L-tyrosine-(FET)-PET may be helpful to improve the definition of radiation target volumes in glioblastomas compared with MRI. We analyzed the relapse patterns in FET-PET after a FET- and MRI-based integrated-boost intensity-modulated radiotherapy (IMRT) of glioblastomas to perform an optimized target volume definition.

**Methods:**

A relapse pattern analysis was performed in 13 glioblastoma patients treated with radiochemotherapy within a prospective phase-II-study between 2008 and 2009. Radiotherapy was performed as an integrated-boost intensity-modulated radiotherapy (IB-IMRT). The prescribed dose was 72 Gy for the boost target volume, based on baseline FET-PET (FET-1) and 60 Gy for the MRI-based (MRI-1) standard target volume. The single doses were 2.4 and 2.0 Gy, respectively. Location and volume of recurrent tumors in FET-2 and MRI-2 were analyzed related to initial tumor, detected in baseline FET-1. Variable target volumes were created theoretically based on FET-1 to optimally cover recurrent tumor.

**Results:**

The tumor volume overlap in FET and MRI was poor both at baseline (median 12 %; range 0–32) and at time of recurrence (13 %; 0–100). Recurrent tumor volume in FET-2 was localized to 39 % (12–91) in the initial tumor volume (FET-1). Over the time a shrinking (mean 12 (5–26) ml) and shifting (mean 6 (1–10 mm) of the resection cavity was seen. A simulated target volume based on active tumor in FET-1 with an additional safety margin of 7 mm around the FET-1 volume covered recurrent FET tumor volume (FET-2) significantly better than a corresponding target volume based on contrast enhancement in MRI-1 with a same safety margin of 7 mm (100 % (54–100) versus 85 % (0–100); *p* < 0.01). A simulated planning target volume (PTV), based on FET-1 and additional 7 mm margin plus 5 mm margin for setup-uncertainties was significantly smaller than the conventional, MR-based PTV applied in this study (median 160 (112–297) ml versus 231 (117–386) ml, *p* < 0.001).

**Conclusions:**

In this small study recurrent tumor volume in FET-PET (FET-2) overlapped only to one third with the boost target volume, based on FET-1. The shrinking and shifting of the resection cavity may have an influence considering the limited overlap of initial and relapse tumor volume. A simulated target volume, based on FET-1 with 7 mm margin covered 100 % of relapse volume in median and led to a significantly reduced PTV, compared to MRI-based PTVs. This approach may achieve similar therapeutic efficacy but lower side effects offering a broader window to intensify concomitant systemic treatment focusing distant failures.

## Introduction

To date, external fractionated radiotherapy is a mainstay in the multimodal treatment strategy of glioblastomas. The diagnostic method of choice for radiation treatment planning is contrast-enhanced MRI owing to its higher anatomical contrast and spatial resolution compared with CT. The differentiation of glioma tissue from surrounding edema, however, may be difficult with MRI and CT particularly when the tumor is not sharply delineated from normal brain tissue, and when the blood-brain barrier (BBB) remains intact [1]. Tumor cells have been detected beyond the margins of contrast enhancement, in the perifocal edema, and even in the adjacent normal-appearing brain parenchyma [2, 3]. Furthermore, after neurosurgical resection BBB disturbances and edema can also be treatment-related and cannot be differentiated from residual tumor or tumor recurrence/progression using conventional MRI [4]. In order to cover all brain areas potentially infiltrated by the tumor, these difficulties lead to rather large target volumes for radiotherapy of glioblastoma [5–9].

In the last decades, amino acid PET using O-(2-^18^F-fluoroethyl)-L-tyrosine (FET) or L-[methyl-^11^C]methionine (MET) have been shown to be particularly useful to determine the extent of cerebral gliomas more precisely than conventional MRI alone [10–15]. Incorporating such molecular or “biological” imaging information has generated the radiooncological concept of the so called “biological target volume” (BTV) [16]. A number of centers have started to integrate amino acid imaging into CT- and MRI-based radiotherapy planning, particularly when high-precision radiotherapy is planned or in the setting of dose escalation studies or for the re-irradiation of recurrent tumors [17–21].

Some studies have examined the recurrence pattern of glioblastoma in relation to the planning target volume (PTV), either based on treatment planning including FET-PET [22], on MET uptake in the baseline study without using PET for planning [23] or based on the localization of tumor recurrence using FET-PET [24]. The matching observation of all these studies was that the recurrences occurred mainly within the PTV. These studies raised the question whether the “in-field”-recurrences can be reduced by dose escalation to the FET-based BTV, e.g., as a stereotactic dose escalation or by means of a simultaneous integrated boost.

In a recent prospective phase-II trial we performed an integrated-boost intensity-modulated radiotherapy (IB-IMRT) with a dose escalation concept giving 72 Gy in 30 fractions to the boost volume based on pre-irradiation ^18^F-FET PET imaging [25]. Compared with historical controls and published MRI-based dose-escalation studies, however, no improvement of progression-free or overall survival could be observed.

Despite this disappointing result, there remains the notion to optimize the irradiation volume using FET PET and thus to possibly reduce side effects. Therefore, we reviewed the follow-up data of the patients in the above-mentioned study in order to analyze the overlap between residual tumor in the baseline FET-PET (FET-1) post-surgery and the relapse tumor volumes as detected also by FET-PET (FET-2). Based on the results different radiation target volumes were simulated in order to achieve optimal coverage of the tumors with minimal irradiation volume.

To the best of our knowledge, this is the first study comparing the tumor volume in FET PET and MRI at the time of radiation treatment planning to that of FET PET and MRI at the time of tumor recurrence.

## Material and methods

### Ethical consideration

This study was approved by the university ethics committee at the RWTH Aachen faculty of medicine (Ref. No. EK027/07). All participants had given written informed consent for their participation in the study and for publication of the data.

### Patients

This retrospective analysis is based on a previous prospective phase II study [25]. In that study, 22 patients with primary glioblastoma (median age, 55 years; range, 36–73 years) were treated with radiotherapy and concomitant temozolomide chemotherapy (RCX) followed by adjuvant temozolomide between 01/2008 and 12/2009 [25]. All patients had pre- and postoperative MRI (T1-, T2- and FLAIR-weighted images) and postoperative FET-PET for radiation treatment planning. The respective MRI- and FET-PET scans, initial (FET-PET1/MRI1) and also at time of relapse (FET-PET2/MRI2), were performed on the same day. Thereafter, all patients were treated with an IB-IMRT.

Within the follow-up time of 15 months (range, 3–34 months) 19 patients presented with tumor recurrence on contrast-enhanced MRI. According to the graduation used by Chan et al. [26], a local, local and distant, and distant only recurrence on MRI was seen in 15, 3, and 1 patient(s), respectively. In 13 patients, a repeated FET-PET scan was done so that MRI and PET data were available both at the time of the planning of radiotherapy and at the time of recurrence. These 13 patients were included in this relapse pattern analysis. Due to a poor medical condition at the time of recurrence, in the remaining 6 patients FET-PET could not be obtained.

### ^18^F-FET PET imaging

The amino acid ^18^F-FET was produced via nucleophilic ^18^F-fluorination with a specific radioactivity of >200 GBq/μmol as described previously. Dynamic PET studies were acquired up to 50 min after intravenous injection of 200 MBq FET in 3-dimensional mode and reconstructed as described previously [27]. The subsequent evaluation was based on the summarized FET-PET data from 20 to 40 min post injection.

### Radiotherapy

The clinical target volumes (CTV) and planning target volume (PTV) were defined as previously described [25]. In brief, a CTV1 was defined from the postoperative FET-PET using an autocontouring process using a tumor-to-brain ratio (TBR) of FET uptake ≥1.6, which is equivalent to the BTV as mentioned above. This cut-off is based on a biopsy-controlled study in cerebral gliomas where a TBR of 1.6 separated best tumoral from peritumoral tissue [14]. Further, a CTV2 was defined as the contrast-enhanced area from pre- and postoperative MRI including a safety margin of 1.5 cm and including the preoperative edema, individually adapted to organs at risk and osseous structures. The PTV1 was based on CTV1 with no additional margin. The PTV2 was generated automatically by adding a 0.5 cm margin around the CTV2. The whole dose was 72 Gy for the PTV1 and 60 Gy for the PTV2 applied with an IB-IMRT (single doses 2.4 and 2 Gy, respectively).

### Analysis of tumor volumes at baseline and at the time of recurrence

In order to analyze the spatial relationship of tumor volumes derived from contrast enhancement in MRI and FET PET at baseline for radiation treatment planning and at the time of recurrence the corresponding data sets were transferred to the Philips Syntegra™ image registration tool. After coregistration of MRI and FET-PET scans the different volumes were compared volumetrically. The contouring and volume analysis was performed using the Philips Pinnacle^3^ treatment planning software (Version 8.0 m, Philips Medical Systems, Eindhoven, NL).

The volume of the tumor showing contrast enhancement of Gd-DTPA on T1-weighted MRI was determined in baseline MRI for radiation treatment planning (MRI-1) and at the time of relapse (MRI-2). Correspondingly, the tumor volume of FET uptake with a TBR ≥ 1.6 was evaluated in the baseline FET PET scan (FET-1) and at the time of recurrence (FET-2).

Intersect tumor volumes of Gd-enhancement in MRI and of increased FET-uptake at baseline (MRI-1 ∩ FET-1) and corresponding intersect at the time of relapse (MRI-2 ∩ FET-2) were determined.

### Analysis of the location of tumor recurrence in relation to PTV1 and PTV2

The primary aim of this study was to analyze the location of the tumor recurrence in FET PET in relation to the tumor area irradiated with a 72 Gy boost (PTV1) which was based on initial FET PET. Furthermore, the recurrence pattern in FET PET in relation to brain area irradiated with a conventional dose of 60 Gy (PTV2) was also considered. This analysis was based on the evaluation of FET-PET data because increased tracer uptake can be considered as a more reliable parameter to determine metabolically active recurrent tumor than contrast enhancement on MRI [28, 29]. For this purpose the tumor volume and fraction of FET positive recurrent tumor within the area irradiated with 60 Gy (PTV2) and within the boost area irradiated with 72 Gy (PTV1) was determined (Table 2).

### Analysis of shifting and shrinking of the resection cavity

The shrinking of the resection cavity was analyzed, measuring the volume of the cavity initial and at time of relapse comparatively. Also, the shifting was analyzed measuring the shift of a manually determined representative center point within the cavity.

### Simulation of the optimal target volume to cover potential relapse areas

Based on FET-PET and MRI at baseline (FET-1 and MRT-1) different target volumes were simulated in order to analyze the coverage of the recurrent tumors in FET-2. Therefore, the surface of baseline tumor volumes in FET-1 and of contrast enhancement in MRI-1 were surrounded by expanded volumes at a distance of 5, 7 and 10 mm to generate different target volumes.

### Statistical analysis

The Wilcoxon test was used to compare the tumor volumes and coverage of recurrent tumor tissue by different simulated PTVs based on FET-PET and contrast-enhanced MRI. The global significance level for the statistical test procedure conducted was chosen as α = 5 %. Statistical analysis was performed using the SPSS Statistics software (Release 20.0, SPSS Inc., Chicago, IL, USA) software.

## Results

### Analysis of tumor volumes at baseline and at the time of recurrence

**Fig. 1:**
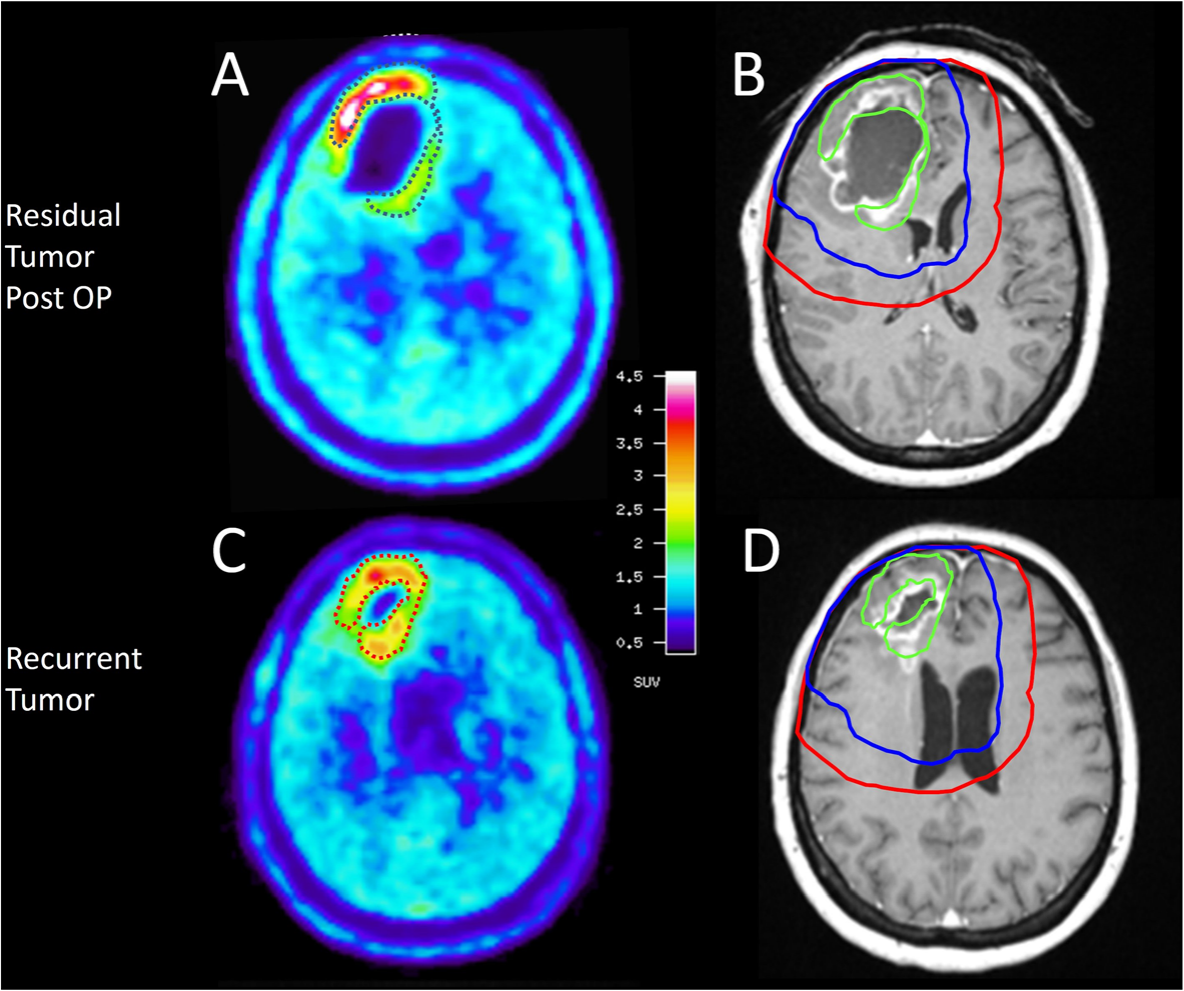
Residual tumor volumes in FET-PET and MRI after glioblastoma resection left frontal are shown in the upper row (**a**, **b**) and of the recurrent tumor in the lower row (**c**, **d**). The tumor volume with increased FET uptake is surrounded by a dotted line in FET-PET (**a**, **c**) and by a green line in contrast-enhanced MRI (**b**, **d**). Note the discrepancy between FET uptake and contrast enhancement both in the baseline scan (**b**) and at the time of relapse (**d**). The definition of PTV2, which is based on MRI, is indicated by the red line (**b**, **d**). The blue line demonstrated a simulated PTV based on a CTV consisting of FET-1 plus 7 mm margin

The tumor volumes for each patient at baseline (FET-1 and MRI-1) and at the time of recurrence (FET-2 and MRI-2) are shown in Table 1. At baseline, the median tumor volume in FET-PET (FET-1) was significantly larger than that of contrast enhancement on MRI-1 (9 (range 1–63) ml vs. 5 (0.6–20) ml; *p* = 0.01) while there was no significant difference between the tumor volumes of FET-PET and MRI at the time of recurrence (FET-2 and MRI-2; 13 (4–67) ml vs. 19 (4–113) ml; *p* = 0.7) The intersect between increased FET uptake (TBR > 1.6) and contrast enhancement in MRI was generally poor both at baseline and at the time of relapse (12 % (0–32) and 13 % (0–100), respectively). The discrepancy between FET uptake and contrast enhancement on MRI is illustrated in Fig. 1b and d.

**Table 1:** Tumor volumes of increased FET-uptake and of Gd-enhancement in MRI at baseline and at time of relapse

Pat. No	Tumor volumes at baseline				Tumor volumes at relapse			
	FET-1 (ml)	MRI-1 (ml)	Intersect FET-1 ∩ MRI-1 (ml)	Intersect FET-1 ∩ MRI-1 (% of FET-1)	FET-2 (ml)	MRI-2 (ml)	Intersect FET-2 ∩ MRI-2 (ml)	Intersect FET-2 ∩ MRI-2 (% of FET-2)
1	10.4	0.8	0.4	3.8	3.7	3.7	0.0	0
2	6.9	0.6	0.2	2.9	10.9	8.9	0.7	8.7
3	6.8	3.7	2.2	32.4	11.3	8.1	5.0	44.2
4	1.1	6.8	1.3	9.9	12.2	9.3	7.6	62.3
5	13.4	6.0	3.5	26.1	13.9	12.0	1.1	7.9
6	15.0	1.3	0.5	3.3	35.1	16.2	23.5	67.0
7	6.7	6.4	0.0	0.0	6.2	19.4	0.8	12.9
8	25.2	1.3	1.3	5.2	22.1	26.8	10.0	45.3
9	4.7	6.5	1.5	31.9	5.6	27.0	5.6	100
10	6.8	4.9	1.6	23.5	56.0	39.6	0.0	0.0
11	62.9	19.4	12.0	19.1	46.7	41.8	23.4	50.1
12	59.0	19.5	11.3	19.2	66.8	43.5	5.5	8.2
13	9.1	1.1	1.1	12.1	12.8	113.0	1.7	13.3
Mean	17.5	6.0	3.0	14.6	23.3	28.4	6.5	32.3
Median	9.1	4.9	1.3	12.1	12.8	19.4	5.0	13.3
SD	19.6	6.1	4.0	11.5	20.9	28.8	8.1	31.5
range	1.1–62.9	0.6–19.5	0–12	0–32.4	3.7–66.8	3.7–113	0–23.5	0-100.0

FET-1: pathological FET-Volume in ml at baseline (post surgery)

MRI-1: Gd-contrast-enhancement in ml at baseline (post surgery)

FET-2: pathological FET-Volume in ml at the time of relapse

MRI-1: Gd-contrast-enhancement in ml at the time of relapse

### Analysis of the location of tumor recurrence in relation to PTV1 and PTV2

Data on the location of pathological tracer uptake in FET PET in relation to PTV1 and PTV2 at the time of tumor recurrence are shown in Table 2. The fraction of the recurrent FET tumor volume within the 72 Gy boost volume PTV1, was only 39 % (12–91), i.e., nearly two thirds of recurrent tumor tissue was located outside the boost volume. In contrast, recurrent FET tumor volume was located to 100 % within the large PTV-2 based on conventional MRI which was irradiated by the standard dose of 60 Gy.

**Table 2:** FET-uptake at time of relapse in relation to PTV-1 and PTV-2

Pat. No	FET Tumor volume at relapse	PTV-1 (72 Gy)		PTV-2 (60 Gy)
	FET-2 (ml)	Part of FET-2 in PTV-1 (ml)	Fraction of FET-2 in PTV-1 (%)	Fraction of FET-2 in PTV-2 (%)
1	3.7	1.2	11.5	100
2	10.9	1.1	15.9	100
3	11.3	3.8	55.9	100
4	12.2	5.1	38.9	100
5	13.9	4.9	36.6	100
6	35.1	13.7	91.3	100
7	6.2	1.6	23.9	100
8	22.1	9.5	37.7	100
9	5.6	0.6	12.8	100
10	56.0	2.8	41.2	100
11	46.7	29.4	46.7	100
12	66.8	29.0	49.2	100
13	12.8	4.4	48.4	100
Mean	23.3	8.2	39.2	100
Median	12.8	4.4	38.9	100
SD	20.9	10.0	21.4	
Range	3.7–66.8	0.6–29.4	11.5–91.3	

FET-2: pathological FET-Volume in ml at the time of relapse

### Analysis of shifting and shrinking of the resection cavity

The resection cavity shrinked by 12 ml (4.8–26) and shifted by 6 mm (1–10.3) in mean over time.

### Simulation of the optimal PTV to cover potential recurrence areas

The target volumes simulated on the basis of FET-PET after resection (FET-1) exhibited generally better coverage of the recurrent FET tumor volume (FET-2) than the corresponding target volumes simulated on the basis of the contrast-enhanced MRI (Table 3). Thus theoretically, a CTV based on FET-1 without any margin showed a significant better coverage of FET-2 than a corresponding target volume based on contrast enhancement in MRI-1 (median 34 % (5–63) vs. 21 % (0–42); *p* < 0.01), FET-1 and MRI-1 with a margin of 5 mm (94 % (42–100) vs. 74 % (0–92); *p* < 0.01), FET-1 and MRI-1 with a margin of 7 mm (100 % (54–100) vs. 85 % (0–100); *p* < 0.01), FET-1 and MRI-1 with a margin of 10 mm (100 % (82–100) vs. 86 % (0–100); *p* < 0.01).

**Table 3:** Coverage of recurrent FET tumor volume by different simulated CTVs based on PET/MRI at baseline

CTV	MRI-1	FET-1	MRI-1	FET-1	MRI-1	FET-1	MRI-1	FET-1
	No margin		+5 mm margin		+7 mm margin		+10 mm margin	
1	0,27	0,33	0,8	0,95	0,87	1,00	0,93	1,00
2	0,08	0,098	0,49	0,61	0,52	0,76	0,7	1,00
3	0,21	0,34	0,61	0,95	0,67	1,00	0,83	1,00
4	0,33	0,42	0,74	0,94	0,85	1,00	0,86	1,00
5	0,25	0,35	0,92	0,96	0,99	1,00	1,00	1,00
6	0,05	0,39	0,19	0,85	0,23	0,92	0,33	0,972
7	0	0,25	0	0,831	0	0,91	0	0,98
8	0,2	0,32	0,76	0,93	1,00	1,00	1,00	1,00
9	0,08	0,11	0,79	0,84	0,88	0,91	0,9	0,95
10	0,16	0,05	0,55	0,42	0,62	0,54	0,84	0,82
11	0,42	0,63	0,51	0,96	0,578	0,97	0,77	1,00
12	0,3	0,44	0,79	1,00	0,90	1,00	0,96	1,00
13	0,22	0,34	0,91	1,00	1,00	1,00	1,00	1,00
mean	0,20	0,31	0,62	0,86	0,70	0,92	0,78	0,98
median	0,21	0,34	0,74	0,94	0,85	1	0,86	1
SD	0,12	0,16	0,27	0,17	0,31	0,13	0,39	0,05
range	0-0,42	0,05-0,63	0-0,92	0,42-1	0-1	0,54-1	0-1	0,82-1

*CTV* Clinical Target Volume

FET-1 pathological FET-Volume in ml at baseline (post surgery)

MRI-1: Gd-contrast-enhancement in ml at baseline (post surgery)

*SD* Standard Deviation

The resulting simulated PTVs on the basis of FET-PET after surgery with different margins in comparison with the actual PTV-2 from the study are shown in Table 4.

**Table 4:** Volumes of standard and simulated PTVs on the basis of FET-PET

Pat. No.	PTV-2 MRI standard (ml)	PTV FET-1 +5 mm margin (ml)	PTV FET-1 +7 mm margin (ml)	PTV FET-1 +10 mm margin (ml)
1	245	122	147	214
2	164	98	118	163
3	117	92	112	143
4	248	125	150	194
5	161	101	114	146
6	256	182	213	286
7	224	106	126	166
8	348	216	253	308
9	209	161	187	259
10	160	138	160	216
11	364	233	263	304
12	386	265	297	342
13	231	175	197	258
mean	240	155	180	230
median	231	138	160	216
SD	83	56	62	67
Range	117–386	92–265	112–297	143–342

(Volumes of conventional PTV2 and simulated PTVs based on FET uptake at baseline (FET-1) expanded by 5, 7 and 10 mm margin in ml. The PTV include an additional margin of 5 mm around the CTV which is standardly used to compensate the set-up- and immobilisation uncertainties)

*PTV* Planning Target Volume

FET-1: pathological FET-Volume in ml at baseline (post surgery)

MRI-1:Gd-contrast-enhancement in ml at baseline (post surgery)

*SD* Standard Deviation

An optimal compromise appears to be a CTV based on FET-1 with a margin of 7 mm because there is a high coverage of recurrent tumor volume in FET-PET (100 % (54–100)) and a significantly smaller PTV compared to a typical MRI-based PTV performed in our study (160 (112–297) ml vs. 231 (117–386) ml, *p* < 0.001).

## Discussion

To date, the definition of the optimal target volume in radiation treatment planning of glioblastomas is controversial [30, 31]. According to current standards, target volume concepts are based on either preoperative or postoperative MRIs, which, however, lead to relative large target volumes [5–9]. PET using radiolabeled amino acids such as FET can offer a more precise delineation of the metabolically active tumor, which is not limited to the area of BBB disruption and is more specific than the information provided by conventional MRI alone [14, 32, 33]. A number of centers have started to integrate the BTV as depicted by amino acid PET into CT- and MRI-based radiotherapy planning [12, 17–20, 24]. Considerable discrepancies between the PTVs arising from MRI and PET have been demonstrated in several studies [12, 17, 19, 24, 34].

In addition to the observed differences in the extent of the tumor in MRI and PET in radiotherapy planning, the localization and the definition of the extent of the recurrent tumor is another diagnostic problem. Treatment-related BBB alterations with consecutive contrast enhancement on conventional MRI can mimic tumor recurrence and are difficult to differentiate from progressive tumor. It has been shown in several studies that FET PET is more reliable to differentiate tumor tissue in recurrent gliomas and posttherapeutic changes in the tissue than conventional MRI [11, 28, 35].

Some studies have examined the recurrence pattern of glioblastoma taking into account amino acid PET in various ways. One study included FET-PET for RT planning but the location of recurrences was evaluated by contrast enhanced MRI only [22]. Another study analyzed the location of recurrences in contrast-enhanced MRI in comparison to MET uptake in the baseline study without using PET for treatment planning [23]. A recent study investigated the localization of tumor recurrence in FET-PET after re-irradiation with bevacizumab in recurrent malignant gliomas [24]. The matching observation of all these studies was that the recurrences occurred mainly within the PTV but it has to be considered that in no study amino acid PET was available in both the baseline study and at the time of relapse.

In this retrospective study we analyzed relapse patterns of glioblastoma in FET-PET and MRI after IB-IMRT with dose escalation based on FET-PET.

A first aspect in this study was the comparison of the extent of contrast enhancement on MRI to that of FET uptake in the baseline study and at the time of recurrence. In agreement with previous studies the intersection between pathological FET uptake and contrast enhancement in MRI was generally poor both at baseline and at the time of recurrence. This observation confirms the view that contrast enhancement in MRI does not reliably reflect the extent of the metabolically active tumor volume and should be therefore considered with caution [12, 17, 19, 24, 34]. Tumor volumes in FET-PET and contrast-enhanced MRI were not significantly different at the time of relapse and the overlap was 13 % in median only.

The comparison of the relapse volume in FET-PET in relation to PTV2 demonstrated that 100 % of the tumor recurrences were located in the routinely performed large target volumes using MRI based treatment planning [5–9]. This is in agreement with the results of previous studies including PET data [22–24] and is also in accordance with the literature based on conventional imaging where all local relapses were detected within the volume enclosed by the 95 % isodosis line of the prescribed dose of 60 Gy [26, 36, 37]. This is not unexpected, since radiation treatment planning based on MRI scans usually encompass the resection cavity and the contrast enhancing area with a margin up to 3 cm [5], resulting in large radiation target volumes.

Comparison of the relapse volume in FET-PET in relation to the boost target volume applied in our study, however, revealed that more than two thirds of recurrent tumor tissue in FET-PET was located outside the boost volume. The limited overlap may be influenced by the shifts of brain tissue due to shrinkage of the resection cavity seen in our analysis (see Fig. 1) but the difference is considerable and cannot be explained solely by these factors. Therefore it can be assumed that a large proportion of recurrences have grown outside the boost volumes i.e. within the area of the prescribed dose of 60 Gy.

Based on this assumption we simulated different CTVs on the basis of FET-PET in order to analyze the coverage of the recurrent tumors in FET-PET. The CTVs simulated on the basis of FET-PET after surgery exhibited generally better coverage of the recurrent FET tumor volume than the corresponding CTVs simulated on the basis of the contrast-enhanced MRI. Using a CTV based on FET-1 with a margin of 7 mm achieved a high coverage of recurrent tumor volume in FET-PET of 100 % (54–100). Accordingly, a significantly smaller PTV results compared to the conventional MR-based PTV used in this study (160 (112–297) ml vs. 231 (117–386) ml, *p* < 0.001). This analysis indicates that a PTV based on FET-PET may achieve a coverage which is at least comparable to standard MRI-based PTVs but less toxic considering the shown PTV reduction. This approach may help to achieve similar therapeutic efficacy but lower side effects. This may be of interest with regard to an intensification of concomitant systemic treatment schemes probably required to improve outcome. Furthermore, sparing of larger parts of the brain increases the systemic treatment options in the case of distant recurrences.

## Conclusion

Overlap of pathological FET uptake in glioblastoma and contrast enhancement in MRI was generally poor both at baseline and at the time of relapse. Relapse volumes of the tumor recurrences in FET-PET were located to 100 % in PTV2 achieving 60 Gy, but more nearly two thirds was located outside the boost volume PTV1. A CTV based on FET with a safety margin of 7 mm covers 100 % of the relapse volume and consecutively reduces the PTV significantly. This approach may achieve similar therapeutic efficacy but lower side effects and offer options to intensify concomitant systemic treatment focusing the problem of distant failures. Because of the small sample size further studies are needed to confirm these findings.

## Ethical consideration and consent to participate

The study was approved by the university ethics committee at the RWTH Aachen faculty of medicine (Ref. No. EK027/07). All participants had given written informed consent for their participation in the study.

## Consent for publication

Not applicable.

## Availability of data and materials

The datasets supporting the conclusions of this article are included within the article.

## Abbreviations

BBB, blood-brain barrier; BTV, biological target volume; CT, computer tomography; CTV, clinical target volume; FET, O-(2-18 F-fluoroethyl)-L-tyrosine; FLAIR, fluid-attenuated inversion recovery; Gd-DTPA, Gadolinium-diethylenetriaminepentacetate; IB-IMRT, integrated boost-intensity-modulated radiotherapy; IMRT, intensity-modulated radiotherapy; MET, L-(methyl-11C)-methionine; MRI, magnetic resonance imaging; PET, positron emission tomography; PTV, planning target volume; RCX, radio-chemotherapy; TBR, tumor-to-brain ratio.
